# Supplementary material for: Efflux pump gene amplifications bypass necessity of multiple target mutations for resistance against dual-targeting antibiotic
Source: Nat Commun. 2023 Jun 9;14:3402. doi: 10.1038/s41467-023-38507-4 (PMC10256781; doi:10.1038/s41467-023-38507-4)

SUPPLEMENTARY INFORMATION

Efflux pump gene amplifications bypass necessity of multiple target mutations for resistance against dual-targeting antibiotic

Kalinga Pavan T. Silva[1], Ganesh Sundar[1], Anupama Khare[1]*

[1]Laboratory of Molecular Biology, National Cancer Institute, National Institutes of Health, Bethesda, MD 20892, USA

*Correspondence: anupama.khare@nih.gov

SUPPLEMENTARY FIGURES AND FIGURE LEGENDS

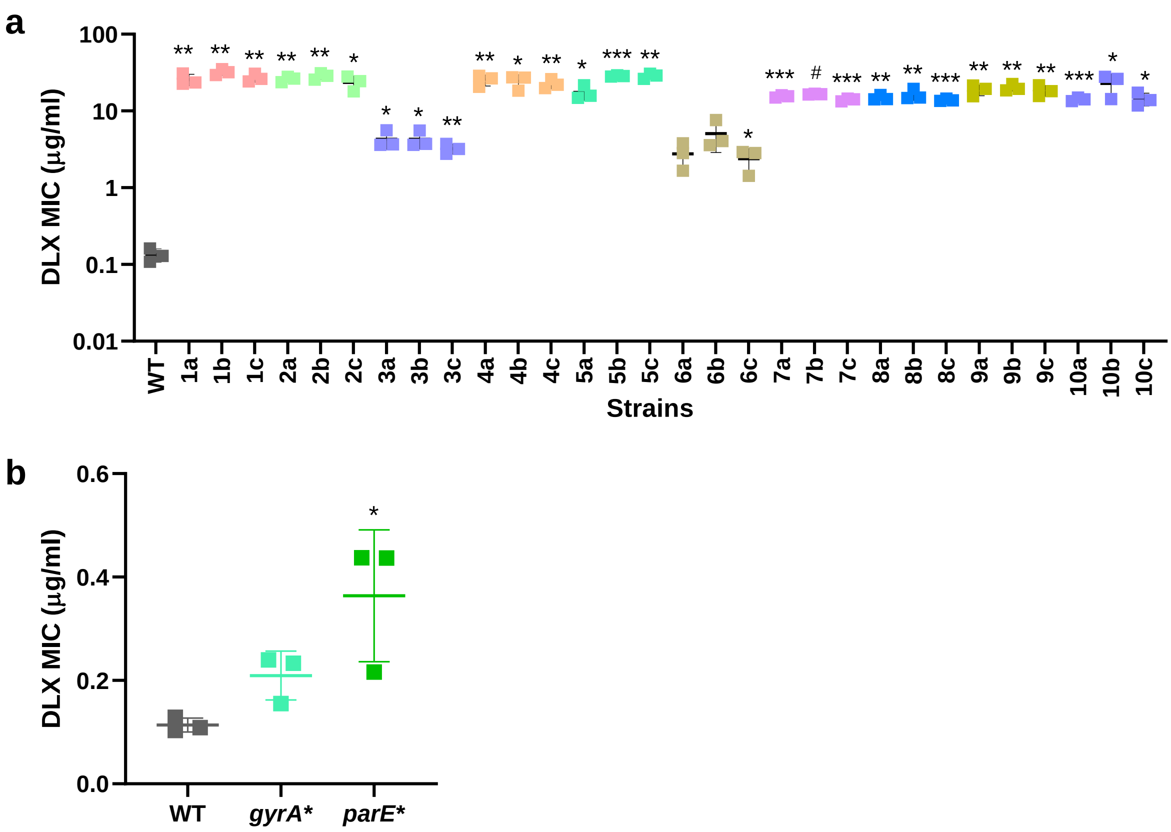

Supplementary Figure 1. Evolved isolates are resistant to delafloxacin (DLX). a DLX MICs of WT, and 3 isolates each from the final passage of all 10 independent populations, tested in MH2. **b** DLX MICs of WT, and allele-replacement strains carrying evolved alleles of *gyrA* (*gyrA*^{E88K}) and *parE* (*parE*^{D432G}) in MH2. Data shown are the mean ± standard deviation of three biological replicates. Significance for all is shown for comparison to the WT, as tested by (**a**) Brown-Forsythe and Welch ANOVA tests, followed by an unpaired t-test with Welch's correction for each comparison and (**b**) a one-way ANOVA with Holm-Sidak's test for multiple comparisons (* $P < 0.05$, ** $P < 0.01$, *** $P < 0.001$, # $P < 0.0001$). For (**a**), P for 1a = 0.0098, 1b = 0.0027, 1c = 0.0049, 2a = 0.0023, 2b = 0.0030, 2c = 0.0155, 3a = 0.0235, 3b = 0.0221, 3c = 0.0077, 4a = 0.0088, 4b = 0.0150, 4c = 0.0064, 5a = 0.0147, 5b = 0.0002, 5c = 0.0019, 6a = 0.0505, 6b =

0.0607, 6c = 0.0428, 7a = 0.0004, 7b < 0.0001, 7c = 0.0006, 8a = 0.0019, 8b = 0.0087, 8c = 0.0005, 9a = 0.0085, 9b = 0.0035, 9c = 0.0085, 10a = 0.0009, 10b = 0.0340, 10c = 0.0122; for (**b**), *P* for *parE** = 0.0163. Source Data are provided in the Source Data file.

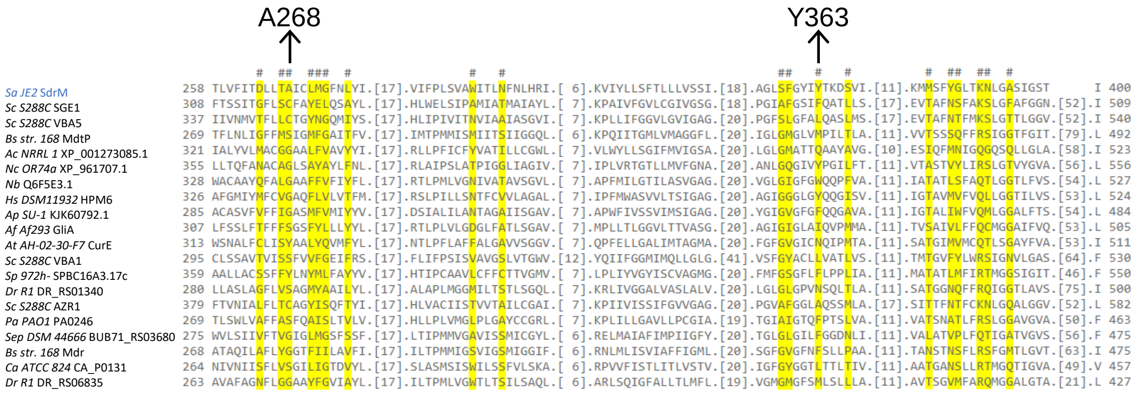

Supplementary Figure 2. A268 and Y363 amino acid residues are predicted to be in the binding pocket of SdrM. The SdrM amino acid sequence was aligned to similar MFS efflux pumps using the Conserved Domain Database[1] (https://www.ncbi.nlm.nih.gov/Structure/cdd/wrpsb.cgi?INPUT_TYPE=precalc&SEQUENCE=447160250). The amino acids predicted to be in the binding pocket are highlighted in yellow, and the SdrM A268 and Y363 residues are indicated. The other protein sequences are from the following species: *Sc*: *Saccharomyces cerevisiae*, *Bs*: *Bacillus subtilis*, *Ac*: *Aspergillus clavatus*, *Nc*: *Neurospora crassa*, *Nb*: *Neocamarosporium betae*, *Hs*: *Hypomyces subiculosus*, *Ap*: *Aspergillus parasiticus*, *Af*: *Aspergillus fumigatus*, *At*: *Aspergillus terreus*, *Sp*: *Saccharomyces pombe*, *Dr*: *Deinococcus radiodurans*, *Pa*: *Pseudomonas aeruginosa*, *Sep*: *Seinonella peptonophila*, *Ca*: *Clostridium acetobutylicum*.

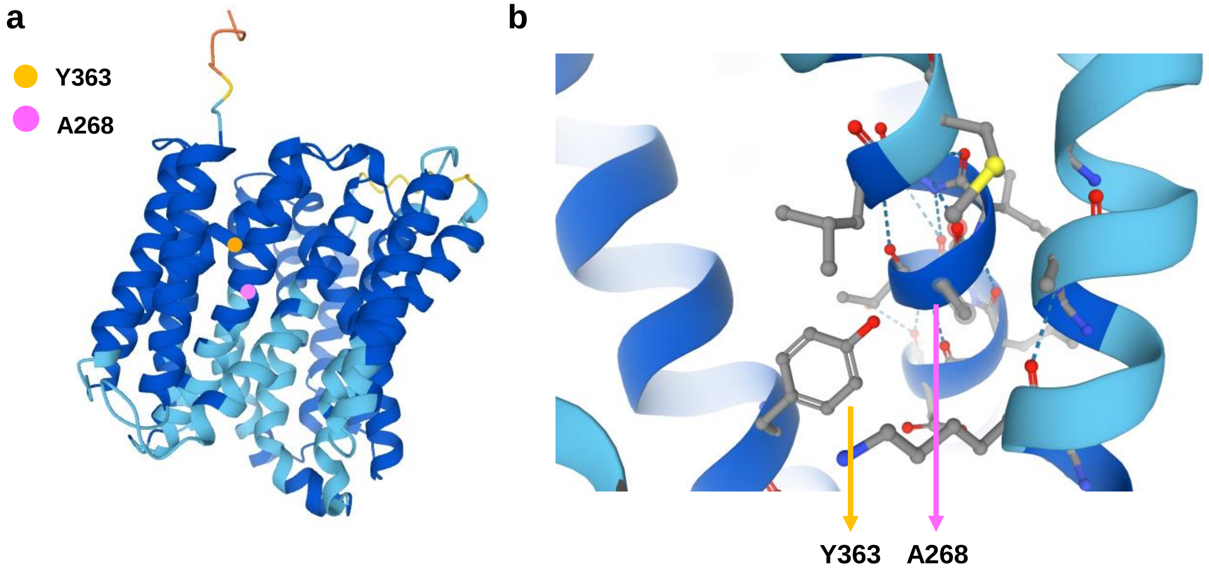

Supplementary Figure 3. The A268 and Y363 residues are predicted to be in close proximity to each other. AlphaFold[2,3] prediction of (**a**) the entire SdrM structure, as well as (**b**) a zoomed-in region containing A268 and Y363.

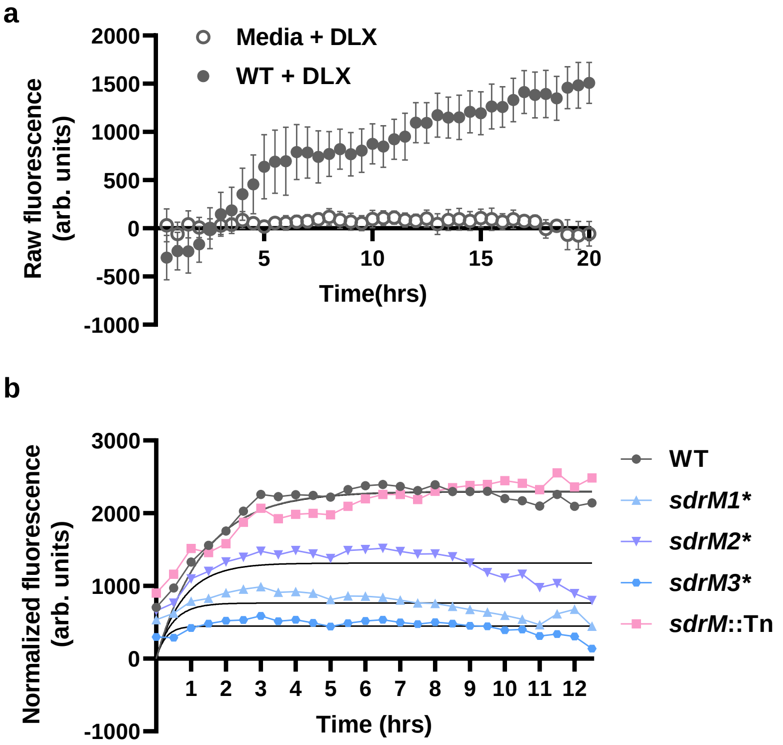

	WT	*sdrM1**	*sdrM2**	*sdrM3**	*sdrM*::Tn
**Rate of efflux (ρ_{out})**	0.3647	1.833	0.9136	3.383	0.3666
**95% confidence interval**	0.3374 to 0.3941	1.628 to 2.085	0.8149 to 1.029	3.023 to 3.828	0.3294 to 0.4075

Supplementary Figure 4. Efflux rates of the allele-replacement strains are higher than the WT. a Raw fluorescence of either WT cells or just the media (M63) with the addition of 0.1 µg/ml DLX. The fluorescence of the respective no DLX condition was subtracted as the background. Data shown are the mean ± SEM of six biological replicates. **b** Normalized fluorescence (intrinsic DLX fluorescence/OD_{600}) was measured for the indicated strains (shown in **Figure 2b**). Least squares fit for the normalized fluorescence with rate of DLX efflux was determined. Shown are the mean values from three biological replicates with the lines representing the best fit curves. The best fit values for rate of efflux are indicated in the table, with the 95% confidence intervals. Source data are provided in the Source Data file.

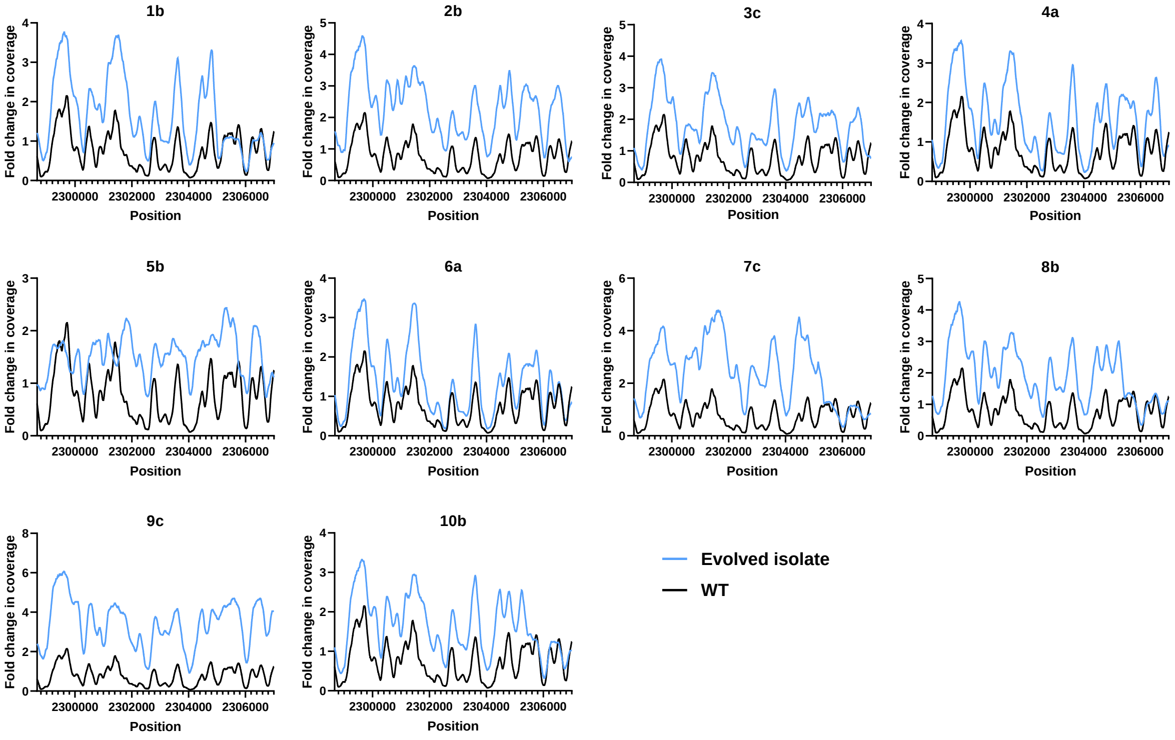

Supplementary Figure 5. Efflux pump gene amplifications were seen in isolates from all evolved populations. Relative read coverage of the amplified region compared to the entire genome shown for the WT, and one isolate from each evolved population. The lines represent a smoothed fit using a generalized additive model considering the nearest 100 neighboring nucleotides.

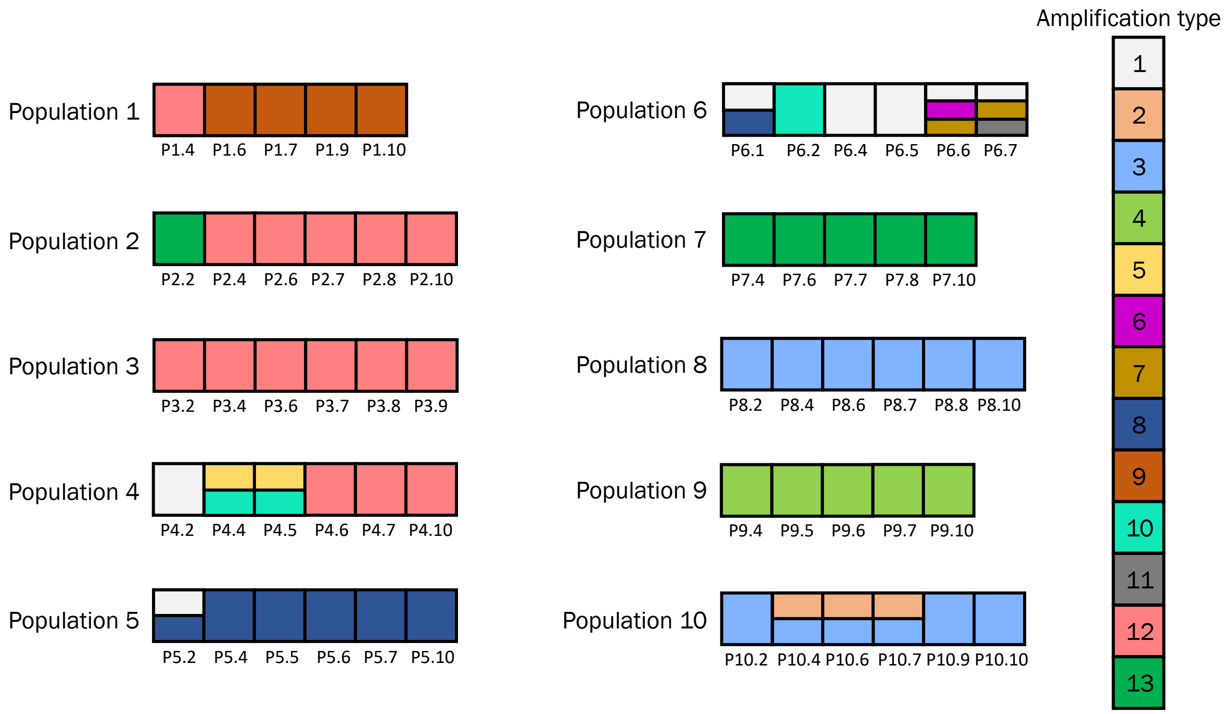

Supplementary Figure 6. The amplifications of the *sdrM* genomic locus are dynamic within the evolving populations. The amplification type(s) present in each sequenced passage from the ten independent populations are shown.

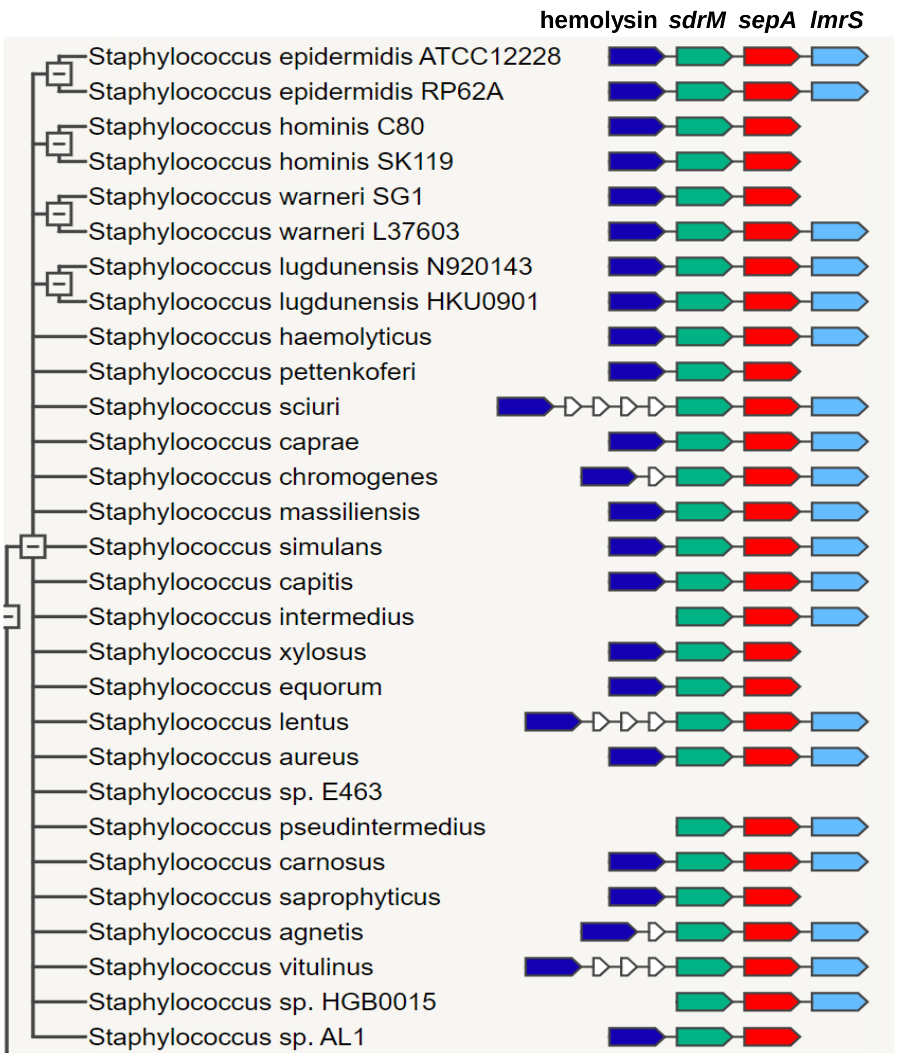

Supplementary Figure 7. The *sdrM*, *sepA*, and *lmrS* genes are located adjacent to each other in most *Staphylococcus* species. Gene neighborhood analysis using the STRING database[4] for genes located near *sdrM* is shown.

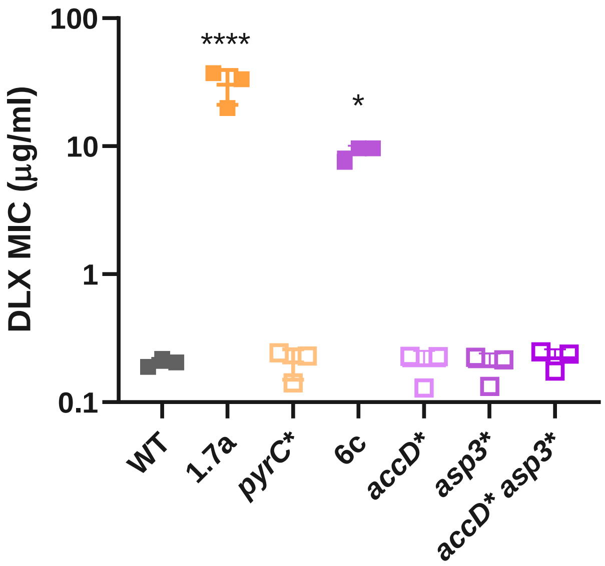

Supplementary Figure 8. Additional mutations in evolved strains do not increase DLX resistance. The DLX MICs of WT, the evolved isolates 1.7a and 6c, and the indicated allelic replacement mutants were measured in M63. Data shown are the mean ± standard deviation of three biological replicates. Significance is indicated for comparison to the WT as tested by a one-way ANOVA with a Dunnett's test for multiple comparisons (* $P = 0.0375$, **** $P < 0.0001$). Source data are provided in the Source Data file.

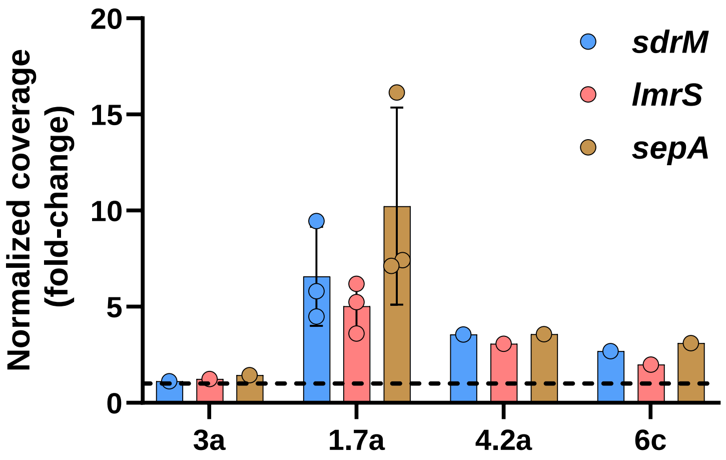

Supplementary Figure 9. Evolved isolates with gene amplifications have high efflux pump copy number. Fold-change of the normalized coverage of the three efflux pump genes *sdrM*, *lmrS*, and *sepA* in the shown evolved isolates. Coverage was normalized to the gene length, and then subsequently to the housekeeping gene *rpoC*. Fold-change is shown compared to the WT. Data shown are the mean ± standard deviation from sequencing three independently prepared libraries (from the same genomic DNA) for 1.7a, and the value from sequencing one library each for the rest of the strains. Source data are provided in the Source Data file.

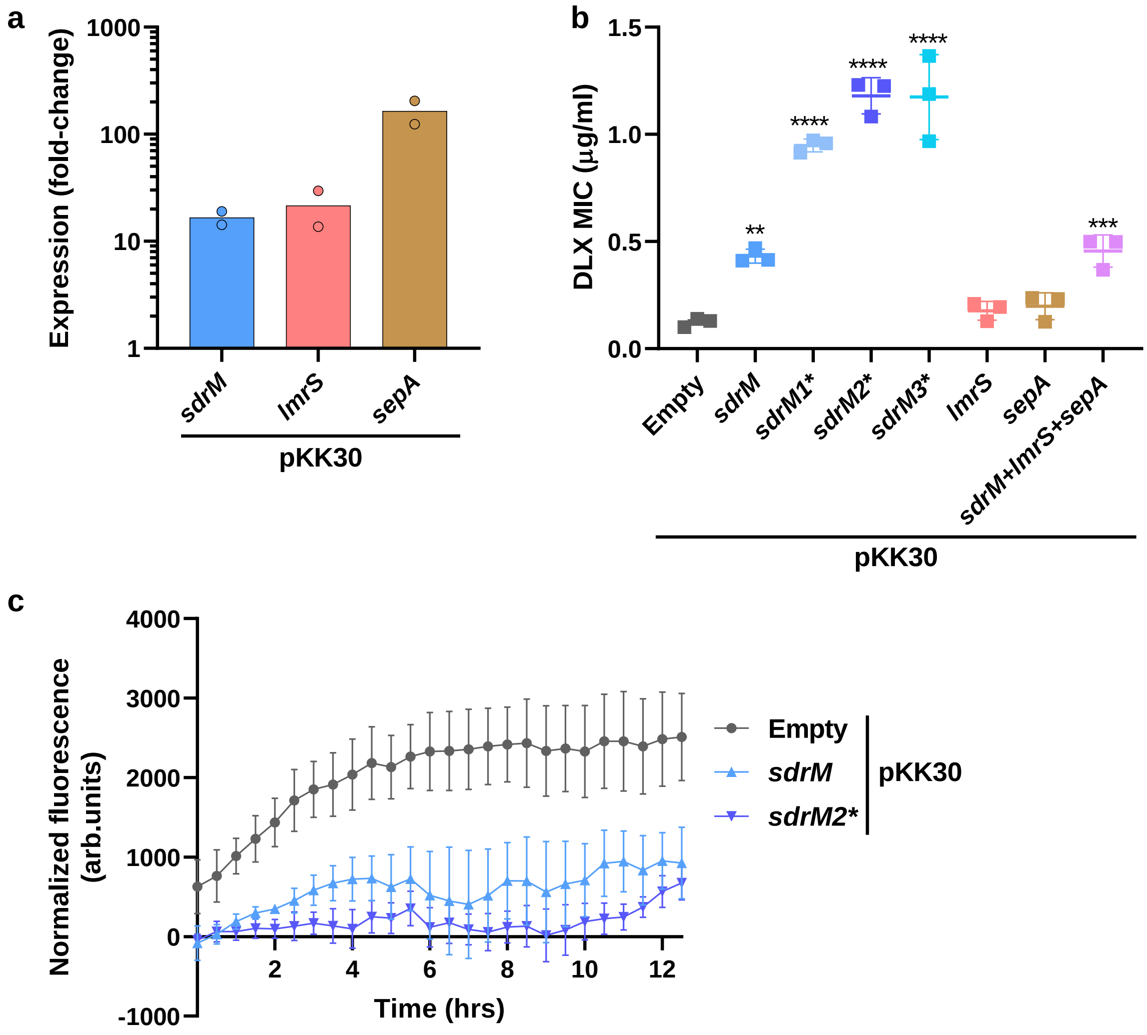

Supplementary Figure 10. Overexpression of *sdrM* increases DLX resistance and efflux. WT strains with either WT or mutant alleles of *sdrM*, *lmrS*, *sepA*, or all three WT efflux pumps expressed from pKK30 under their native promoters, were tested for expression, DLX resistance, and efflux. **a** Expression of the indicated pumps in the respective over-expression strains was measured by RT-qPCR. Data is shown as fold-change in expression compared to a WT strain carrying the pKK30 empty plasmid. Data shown are the mean of two biological replicates. **b** DLX MICs were measured in M63. Data shown are the mean ± standard deviation of three biological replicates. Significance is shown for comparison to the strain with the empty pKK30 plasmid, as

tested by a one-way ANOVA with Holm-Sidak's test for multiple comparisons (** $P = 0.0015$, *** $P = 0.0010$, **** $P < 0.0001$). **c** Normalized fluorescence (intrinsic fluorescence of DLX / OD_{600}) was measured for the indicated strains. Data shown are the mean ± standard error of three biological replicates. Source data are provided in the Source Data file.

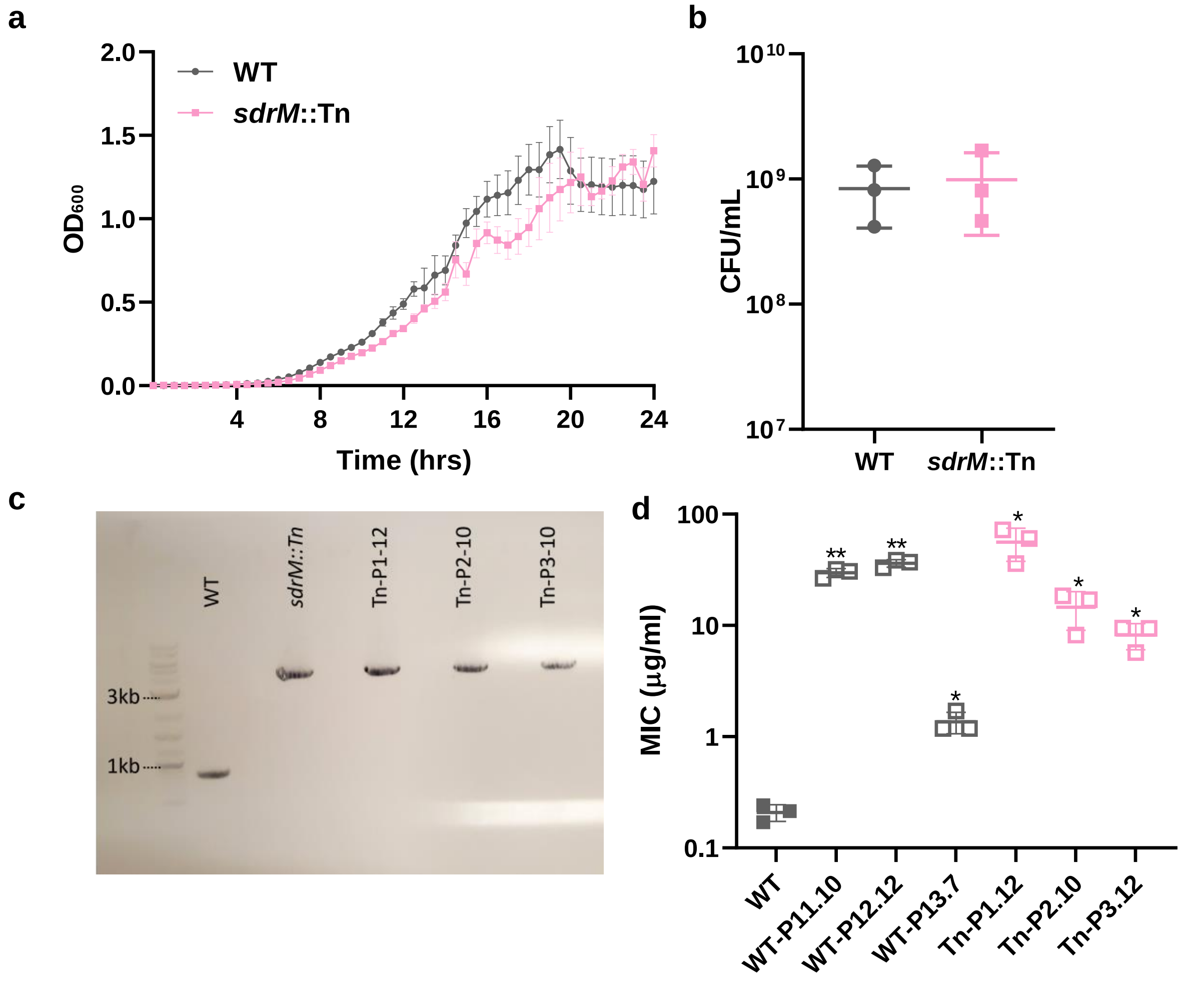

Supplementary Figure 11. The *sdrM*::Tn mutant is stable, has similar growth characteristics to the WT, and evolves high DLX resistance. a OD_{600} measurements of the WT and *sdrM*::Tn strains diluted 1:2500 from overnight cultures into fresh M63 media. Data shown are the mean ± standard error from six biological replicates. **b** Cell density of overnight grown WT and *sdrM*::Tn strains was determined as CFU/mL. Data shown are the mean ± standard deviation from three biological replicates. **c** Gel showing the bands from a PCR using primers flanking the transposon insertion site of *sdrM*::Tn. The WT shows a band of ~800 bp, while *sdrM*::Tn and populations from the terminal passage of its three independently evolved populations show only one higher band of ~4 kb, indicating the *sdrM* allele containing the

inserted transposon. **d** DLX MICs of WT, and populations from the terminal passages of the three independently evolved WT and *sdrM*::Tn populations. Data shown are the mean ± standard deviation of three biological replicates. Significance is shown for comparison to the WT, as tested by Brown-Forsythe and Welch ANOVA tests, followed by an unpaired t-test with Welch's correction for each comparison. (* $P < 0.05$, ** $P < 0.01$, P for WT-P11.10 = 0.0029, WT-P12.12 = 0.0021, WT-P13.7 = 0.0204, Tn-P1.12 = 0.0344, Tn-P2.10 = 0.0466, Tn-P3.12 = 0.0234). Source data for (**a, b, d**) are provided in the Source Data file, and the uncropped scan of (**c**) is provided at the end of this file.

Supplementary Table 1. Strains and plasmids used in this study.

Name	Description	Source
**Strains**		
S. aureus		
JE2	*Staphylococcus aureus* subsp. *aureus* USA300_FPR3757 (CA-MRSA)-JE2	[5]
CF001	AMT0150-13; age: 8.05 years	CFF Isolate Core
CF106	AMT0538-04; age: 3.92 years	CFF Isolate Core
RN4220	Restriction modification deficient *S. aureus*; used to shuttle pKK30 derivatives into JE2	[6-8]
SB229	JE2 *sdrM1**	This study
SB225	JE2 *sdrM2**	This study
SB262	JE2 *sdrM3**	This study
SB267	JE2 *gyrA**	This study
SB268	JE2 *parE**	This study
SB236	JE2 *pyrC**	This study
SB224	JE2 *accD**	This study
SB234	JE2 *asp3**	This study
SB235	JE2 *accD* asp3**	This study
NE531	JE2 *sdrM::*Tn	[9]
E. coli		
DH5α-λpir	DH5α lysogenized with λpir; host for pKK30	[10]
IM08B	For plasmid transfer into *S. aureus*	[11]
**Plasmids**		
pIMAY*	*S. aureus* allelic exchange plasmid; Cm^R	[12]
pKK30	Expression vector for *S. aureus*; Tmp^R	[13]
pSB271	pKK30-*sdrM*	This study
pSB273	pKK30-*sdrM1**	This study
pSB272	pKK30-*sdrM2**	This study
pSB324	pKK30-*sdrM3**	This study
pSB274	pKK30-*lmrS*	This study
pSB275	pKK30-*sepA*	This study
pSB414	pKK30-*sdrM-sepA-lmrS*	This study

Cm^R: chloramphenicol resistant; Tmp^R: trimethoprim resistant

Supplementary Table 2. Primers used in this study.

Primer Description	Primer Sequence*
Amplify *sdrM* for pIMAY* F	GGTATCGATAAGCTTGATATCGAATTCGAAGGAGGTATTTCATGCGA
Amplify *sdrM* for pIMAY* R	ATTGGAGCTCCACCGCGGTGGCGGCCGCCGCACCAGAAAGTACAAAAA
Check pIMAY*-*sdrM* integration F	AATGAAAAGTCGCGCCTCTA
Check pIMAY*-*sdrM* integration R	AAAAGCTGGGGGAAACTCCG
Sanger sequencing for *sdrM1** and *sdrM2**	ATAAGCCCAATGCTAATTAC
Amplify *accD** for pIMAY* F	ATCGATAAGCTTGATATCGTGCGCTGTCACAAGATATG
Amplify *accD** for pIMAY* R	CTCCACCGCGGTGGCTCCACATCATTTTTATCTTGAGATTC
Check pIMAY*-*accD* integration F	TCTAGGGTCGTAGGTCTTTC
Check pIMAY*-*accD* integration R	GCAATTTTTGAAGGACGTTCATA
Sanger sequencing for *accD*	CAGCAACGCCAAATTTCATA
Amplify *asp3** for pIMAY* F	TGGAGCTCCACCGCGGTGGCCATTTCAAGAGCTATTACCTG
Amplify *asp3** for pIMAY* R	TATCGATAAGCTTGATATCGTTTAACTTCATCGCTCCATG
Check pIMAY*-*asp3* integration F	AGAAGAGTTCGGTACAGCATTAG
Check pIMAY*-*asp3* integration R	CTCGCTTCGCTAAATAATCATTC
Sanger sequencing for *asp3**	CGAGCCAAACATGACCAAAC
Amplify *pyrC** for pIMAY* F	TGGAGCTCCACCGCGGTGGCCGTAGTAATTACCATAGTTTAAAAGC
Amplify *pyrC** for pIMAY* R	TATCGATAAGCTTGATATCGCCTAAACGGTAGCCTTCG
Check pIMAY*-*pyrC* integration F	CCAATTGCGAATGCTGGTG
Check pIMAY*-*pyrC* integration R	GATCTGACCTGTATATGATGGATC
Sanger sequencing for *pyrC**	CCAATTGCGAATGCTGGTG
Amplify *sdrM3** for pIMAY* F	TGGAGCTCCACCGCGGTGGCTGATAGGCGGTGACTATG
Amplify *sdrM3** for pIMAY* R	TATCGATAAGCTTGATATCGCTAGTATCTAGGAAATTTATTATTTC
Check pIMAY*-*sdrM3* integration F and Sanger sequencing for *sdrM3**	TGTCACGTTACAAGTTCCTAGAG
Check pIMAY*-*sdrM3* integration R	CCATTAAATGCTCGACTGCAAATA
Amplify *gyrA* for pIMAY* F	TATCGATAAGCTTGATATCGCACGATACAAAGGTCTTG
Amplify *gyrA* for pIMAY* R	TGGAGCTCCACCGCGGTGGCTAGCATAAAAATAAGACTCCC
Check pIMAY*-*gyrA* integration F	TCTGAATTGAATCCAACACCAA
Check pIMAY*-*gyrA* integration R	GTGCCACCATCAAGACTTATCA
Sanger sequencing for *gyrA**	GAACTGAACTTTTGAAGGAG
Amplify *parE** for pIMAY* F	TATCGATAAGCTTGATATCGAGAATAACTATTGTATAGTTTTAAAAACG
Amplify *parE** for pIMAY* R	TGGAGCTCCACCGCGGTGGCATCACCTAAAACATCTTCAAG
Check pIMAY*-*parE* integration F	GGGAAAGCGCCGATAAGATA
Check pIMAY*-*parE* integration R	AGTCTCCATGTGGATGATATTG
Sanger sequencing for *parE**	TGAAGCTAGAAGTGCTGTTGAT

Gibson assembly for pKK30 F	GCGGCCGCTAGCCTAGGAGC
Gibson assembly for pKK30 R	ATCGCCTGTCACTTTGCTTGATATATGA
Amplify *sdrM* for pKK30 F	ATCAAGCAAAGTGACAGGCGATACAGTATTTATTTTATTATGGGGAAC
Amplify *sdrM* for pKK30 R	GAGCTCCTAGGCTAGCGGCCGCCTATTCTTTTGATTGAGATGAC
Amplify *sepA* for pKK30 F	ATCAAGCAAAGTGACAGGCGATAATGATGTCATTTTATGGATTAAC
Amplify *sepA* for pKK30 R	GAGCTCCTAGGCTAGCGGCCGCCTATTTTCTATTATTTAAATTTTTAC
Amplify *lmrS* for pKK30 F	ATCAAGCAAAGTGACAGGCGATTCAATATCGATTTTTGGGTC
Amplify *lmrS* for pKK30 R	GAGCTCCTAGGCTAGCGGCCGCTTAAAATTTCCTTCTATTACTTTC
Sanger sequencing for pKK30 F	CTGGGAAGTCGAATCTTCAGTAG
Sanger sequencing for pKK30 R	ATGATAGGTCTGGCAAAGCC
qPCR for *rpoC* F	CTGTGAAAGAATTTTCGGAC
qPCR for *rpoC* R	CTTTCACGACGTACTTTAGA
qPCR for *sdrM* F	GCAATGATCGCAATCGGTAT
qPCR for *sdrM* R	GGCATAGTTGGCAGTGTTTG
qPCR for *lmrS* F	TGCGATGGCGATGTAGATAAA
qPCR for *lmrS* R	CTCACATGGCACGGCTATTA
qPCR for *sepA* F	CCATGATGACCCAAAAATCG
qPCR for *sepA* R	TTAGAGGCGCGACTTTTCAT
Check Amplification 1 F	TATGCCTCCTGCTGAGTTTG
Check Amplification 1 R	CCTTGGTCAAGCGGTTAAGA
Check Amplification 2,9 F	TGTATAAGACACACCACCTAAGAAA
Check Amplification 2 R	GCTATGTGTGGACGGGATAAG
Check Amplification 3 R	CTTGACTGCGAGACCTACAA
Check Amplification 4 F	GAAGTGCGTATTCAATGGAGAGT
Check Amplification 4 R	CTAGCTGTGTTGGCTTTCT
Check Amplification 5,6 F	ATCGCATGAAATACCTCCTTCT
Check Amplification 5 R	TGGGATACTACCCTAGCTGTG
Check Amplification 7 F	CTCGAAAGTAATTCGCCCACTA
Check Amplification 6,7 R	AGAAGAGCCGCAGTGAATAG
Check Amplification 8 F	CGTAAAGGTTCGATGTCCAAAG
Check Amplification 8 R	GGAGTCAGAACATGGGTGATAAG
Check Amplification 10 F	GCACTTTCAGGTACCTCCTTAG
Check Amplification 11 F	TCCATAATACGACCCTGTTGATTTA
Check Amplification 9,10,11,12 R	GCGACTTTCTGGTCTGTAACT
Check Amplification 12 F	ACCTTAAACCTTCTGCCAATCT
Check Amplification 3,13,14,15 F	ACGTAAATCTTCTGTTGCAGTTG
Check Amplification 13 R	GCGGCGTGCCTAATACAT
Check Amplification 14, 15 R	GCTGGATCACCTCCTTTCTAAG
Check Amplification 16 F	GTGTTGGGTCCCTTCGTATAAT
Check Amplification 16 R	TGCAATAGCCTCCGGTAAAG
Check *sdrM*::Tn F	GAATCCCATTAAACAAATAGC
Check *sdrM*::Tn R	ATGCGATTAAAGTCAATCAT

***** Underlined sequences represent homology to the respective plasmid for Gibson assembly.

References

1 Lu, S. *et al.* CDD/SPARCLE: the conserved domain database in 2020. *Nucleic Acids Research* **48**, D265-d268 (2020).

2 Jumper, J. *et al.* Highly accurate protein structure prediction with AlphaFold. *Nature* **596**, 583-589 (2021).

3 Varadi, M. *et al.* AlphaFold Protein Structure Database: massively expanding the structural coverage of protein-sequence space with high-accuracy models. *Nucleic Acids Research* **50**, D439-d444 (2022).

4 Szklarczyk, D. *et al.* STRING v11: protein–protein association networks with increased coverage, supporting functional discovery in genome-wide experimental datasets. *Nucleic Acids Research* **47**, D607-D613 (2019).

5 Kennedy, A. D. *et al.* Epidemic community-associated methicillin-resistant *Staphylococcus aureus*: recent clonal expansion and diversification. *Proceedings of the National Academy of Sciences* **105**, 1327-1332 (2008).

6 de Azavedo, J. C. *et al.* Expression of the cloned toxic shock syndrome toxin 1 gene (tst) in vivo with a rabbit uterine model. *Infection and Immunity* **50**, 304-309 (1985).

7 Fairweather, N., Kennedy, S., Foster, T. J., Kehoe, M. & Dougan, G. Expression of a cloned *Staphylococcus aureus* alpha-hemolysin determinant in *Bacillus subtilis* and *Staphylococcus aureus*. *Infection and Immunity* **41**, 1112-1117 (1983).

8 Kreiswirth, B. N. *et al.* The toxic shock syndrome exotoxin structural gene is not detectably transmitted by a prophage. *Nature* **305**, 709-712 (1983).

9 Fey, P. D. *et al.* A genetic resource for rapid and comprehensive phenotype screening of nonessential *Staphylococcus aureus* genes. *mBio* **4**, e00537-00512 (2013).

10 Dunn, A. K., Martin, M. O. & Stabb, E. V. Characterization of pES213, a small mobilizable plasmid from *Vibrio fischeri*. *Plasmid* **54**, 114-134 (2005).

11 Monk, I. R., Tree, J. J., Howden, B. P., Stinear, T. P. & Foster, T. J. Complete bypass of restriction systems for major *Staphylococcus aureus* lineages. *mBio* **6**, e00308-00315 (2015).

12 Schuster, C. F., Howard, S. A. & Gründling, A. Use of the counter selectable marker PheS* for genome engineering in *Staphylococcus aureus*. *Microbiology* **165**, 572-584 (2019).

13 Krute, C. N. *et al.* Generation of a stable plasmid for in vitro and in vivo studies of *Staphylococcus* species. *Applied and Environmental Microbiology* **82**, 6859-6869 (2016).

Uncropped scan of Supplementary Figure 11c

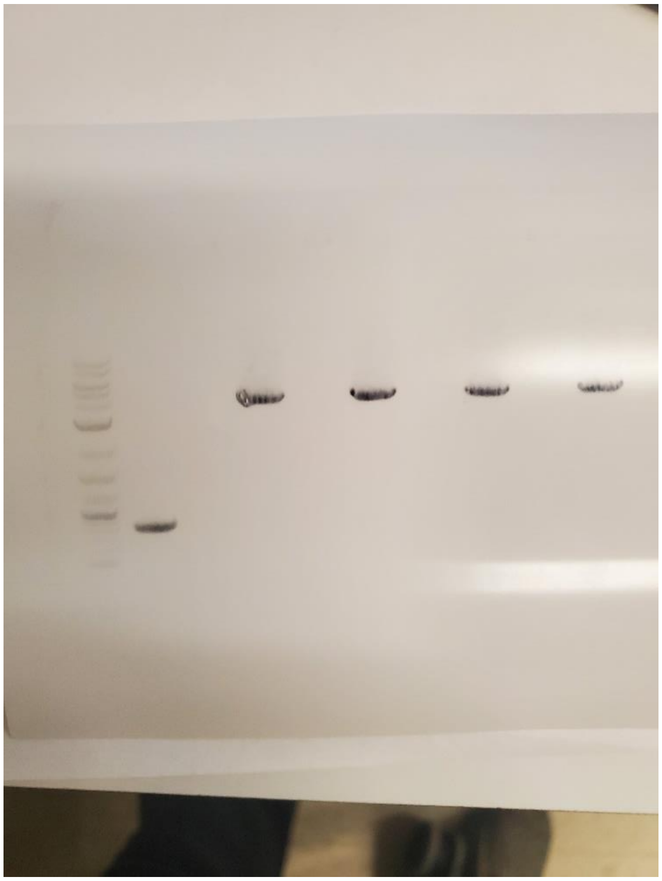

Supplement: Supplementary file 1 — Supplementary Information [file 41467_2023_38507_MOESM1_ESM.pdf]
