# Supplementary material for: Efflux pump gene amplifications bypass necessity of multiple target mutations for resistance against dual-targeting antibiotic
Source: Nat Commun. 2023 Jun 9;14:3402. doi: 10.1038/s41467-023-38507-4 (PMC10256781; doi:10.1038/s41467-023-38507-4)
Supplement: Supplementary file 3 — Description of Additional Supplementary Files [file 41467_2023_38507_MOESM3_ESM.pdf]

Description of Additional Supplementary Files

Supplementary Dataset 1. Description of the passages of evolution of the DLX resistant populations and isolates.

Supplementary Dataset 2. Mutations and amplifications present in the evolved populations and isolates.

Supplementary Dataset 3. Allelic diversity of the SdrM protein sequence in publicly available *S. aureus* genomes.

Supplementary Dataset 4. Details of the sixteen different amplification types of *sdrM* seen in the evolved populations and isolates.

Supplementary Dataset 5. MLST and SCCmec typing of the two clinical isolates.
