# Supplementary material for: Efflux pump gene amplifications bypass necessity of multiple target mutations for resistance against dual-targeting antibiotic
Source: Nat Commun. 2023 Jun 9;14:3402. doi: 10.1038/s41467-023-38507-4 (PMC10256781; doi:10.1038/s41467-023-38507-4)
Supplement: Supplementary file 9 — Reporting Summary [file 41467_2023_38507_MOESM9_ESM.pdf]

Corresponding author(s): Anupama Khare
Last updated by author(s): Apr 27, 2023

Reporting Summary

Nature Portfolio wishes to improve the reproducibility of the work that we publish. This form provides structure for consistency and transparency in reporting. For further information on Nature Portfolio policies, see our Editorial Policies and the Editorial Policy Checklist.

Statistics

For all statistical analyses, confirm that the following items are present in the figure legend, table legend, main text, or Methods section.

n/a	Confirmed	
☐	☒	The exact sample size (*n*) for each experimental group/condition, given as a discrete number and unit of measurement
☐	☒	A statement on whether measurements were taken from distinct samples or whether the same sample was measured repeatedly
☐	☒	The statistical test(s) used AND whether they are one- or two-sided<br>*Only common tests should be described solely by name; describe more complex techniques in the Methods section.*
☐	☒	A description of all covariates tested
☐	☒	A description of any assumptions or corrections, such as tests of normality and adjustment for multiple comparisons
☐	☒	A full description of the statistical parameters including central tendency (e.g. means) or other basic estimates (e.g. regression coefficient) AND variation (e.g. standard deviation) or associated estimates of uncertainty (e.g. confidence intervals)
☐	☒	For null hypothesis testing, the test statistic (e.g. *F*, *t*, *r*) with confidence intervals, effect sizes, degrees of freedom and *P* value noted<br>*Give P values as exact values whenever suitable.*
☒	☐	For Bayesian analysis, information on the choice of priors and Markov chain Monte Carlo settings
☒	☐	For hierarchical and complex designs, identification of the appropriate level for tests and full reporting of outcomes
☒	☐	Estimates of effect sizes (e.g. Cohen's *d*, Pearson's *r*), indicating how they were calculated

Our web collection on statistics for biologists contains articles on many of the points above.

Software and code

Policy information about availability of computer code

Data collection	The optical density and fluorescence measurements were performed in a Biotek Synergy H1 microplate reader using the Gen5 software v3.09 and v3.11.
Data analysis	For analysis of the whole genome sequencing data, the Illumina adapters were removed using cutadapt v4.0, the reads were trimmed with trimmomatic v0.39, or both steps done using fastp v0.23.2, and the mutations in the evolved populations and isolates were identified using breseq v0.37.1. The genomes of the clinical isolates CF001 and CF106 were assembled using Unicycler v0.4.8 on PATRIC (now integrated into the Bacterial and Viral Bioinformatics Resource Center). The assembled contigs were then annotated using Prokka v1.14.6. These annotated genomes were used as a reference to identify mutations in the respective evolved populations using breseq.<br>MLST of CF001 and CF106 was done using the PubMLST website online (pubmlst.org), and the SCCmec typing was done using Staphopia-sccmec.<br>Analysis of the allelic variation in SdrM was done using NCBI blast v2.13.0+.

For manuscripts utilizing custom algorithms or software that are central to the research but not yet described in published literature, software must be made available to editors and reviewers. We strongly encourage code deposition in a community repository (e.g. GitHub). See the Nature Portfolio guidelines for submitting code & software for further information.

Data

Policy information about availability of data

All manuscripts must include a data availability statement. This statement should provide the following information, where applicable:

- Accession codes, unique identifiers, or web links for publicly available datasets
- A description of any restrictions on data availability
- For clinical datasets or third party data, please ensure that the statement adheres to our policy

Genome assemblies of S. aureus isolates to analyze allelic variability of SdrM were downloaded from the NCBI Pathogen Detection database (https://www.ncbi.nlm.nih.gov/pathogens/). The whole-genome sequencing data from this study have been deposited at the NCBI Short Read Archive (SRA), associated with the BioProject PRJNA904786 (https://www.ncbi.nlm.nih.gov/bioproject/PRJNA904786/). Source data for all figures are provided with his paper.

Research involving human participants, their data, or biological material

Policy information about studies with human participants or human data. See also policy information about sex, gender (identity/presentation), and sexual orientation and race, ethnicity and racism.

Reporting on sex and gender	Not applicable
Reporting on race, ethnicity, or other socially relevant groupings	Not applicable
Population characteristics	Not applicable
Recruitment	Not applicable
Ethics oversight	Not applicable

Note that full information on the approval of the study protocol must also be provided in the manuscript.

Field-specific reporting

Please select the one below that is the best fit for your research. If you are not sure, read the appropriate sections before making your selection.

☒ Life sciences ☐ Behavioural & social sciences ☐ Ecological, evolutionary & environmental sciences

For a reference copy of the document with all sections, see nature.com/documents/nr-reporting-summary-flat.pdf

Life sciences study design

All studies must disclose on these points even when the disclosure is negative.

Sample size	Sample sizes were chosen based on the accepted standards in the field - typically the sample size was between 3-5. For the original evolution experiment, 10 independent populations were used to allow for detection of less common evolutionary paths leading to resistance. For the experiments measuring evolvability, 12 independent populations were used to allow for measurement of evolution frequencies between ~10-100%.
Data exclusions	No data were excluded from the analyses.
Replication	All attempts at replication were successful.
Randomization	Randomization is not relevant to the study as the experiments were conducted with defined strains/mutants, under standardized conditions. Replicate experiments were conducted under identical conditions.
Blinding	Blinding was not relevant to the study as the data collection and measurements are quantitative (and not subjective), and therefore not subject to human bias.

Reporting for specific materials, systems and methods

We require information from authors about some types of materials, experimental systems and methods used in many studies. Here, indicate whether each material, system or method listed is relevant to your study. If you are not sure if a list item applies to your research, read the appropriate section before selecting a response.

Materials & experimental systems

n/a	Involved in the study
☒	☐ Antibodies
☒	☐ Eukaryotic cell lines
☒	☐ Palaeontology and archaeology
☒	☐ Animals and other organisms
☒	☐ Clinical data
☒	☐ Dual use research of concern
☒	☐ Plants

Methods

n/a	Involved in the study
☒	☐ ChIP-seq
☒	☐ Flow cytometry
☒	☐ MRI-based neuroimaging
